# Postnatal *Syk *deletion in mice clarifies the function of Syk in an anti-collagen antibody-induced arthritis model

**DOI:** 10.1186/ar3577

**Published:** 2012-02-09

**Authors:** Naoko Ozaki, Shinobu Suzuki, Hiromitsu Hara, Hiroki Yoshida

**Affiliations:** 1 Division of Molecular and Cellular Immunoscience, Department of Biomolecular Sciences, Faculty of Medicine, Saga University, Nabeshima, Saga, 849-8501, Japan; 2 Department of Molecular & Cellular Biology, Kobe Pharma Research Institute, Nippon Boehringer Ingelheim Co Ltd, Minatojima-Minamimachi, Chuo-ku, Kobe, Hyogo, 650-0047, Japan

## 

**Figure 1:**
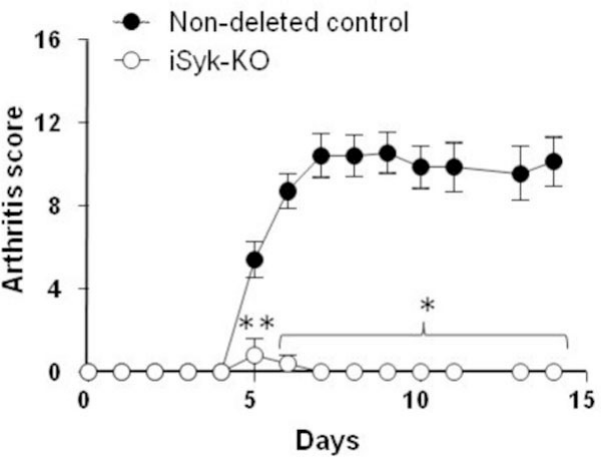
**Arthritis development in iSyk KO mice**. Arthritis was induced by i.p. administration of anti-collagen Ab followed by LPS. Arthritis score was monitored. *; *P *< 0.001, **; *P *< 0.01.

Spleen tyrosine kinase (Syk) is a cytoplasmic protein expressed mainly in immune cells including macrophages and neutrophils and is associated with receptors containing an immunoreceptor tyrosine-based activation motif (ITAM), such as Fcγ receptors. As Syk-mediated signaling plays an important role in activation of immune responses, to investigate whether specific interruption of Syk-mediated signaling can affect the development of rheumatoid arthritis (RA), we used tamoxifen-induced conditional Syk-KO mice (iSyk KO) to evaluate the importance of Syk on disease development. Using a collagen antibody-induced arthritis model (CAIA), iSyk KO mice showed significantly attenuated disease severity compared to Syk non-deleted mice (Figure 1). Although iSyk KO mice contained reduced B cell numbers after deletion of Syk in adulthood, B cells are not required for arthritis development in CAIA, as demonstrated by using muMT mice which lack B cells. On the other hand, Syk-deficient macrophages produced less MCP-1 and IL-6 than Syk-sufficient cells after FcR ligation, which can account for the absence of a pronounced accumulation of neutrophils and macrophages in the joints of iSyk KO mice. Our results demonstrate that Syk in macrophages is likely a key player in antibody-induced arthritis, mediating the release of pro-inflammatory cytokines and chemokines after macrophages bind anti-collagen antibody, and indicate that Syk is a promising target for arthritis therapy.

